# Bystander autophagy mediated by radiation-induced exosomal miR-7-5p in non-targeted human bronchial epithelial cells

**DOI:** 10.1038/srep30165

**Published:** 2016-07-15

**Authors:** Man Song, Yu Wang, Zeng-Fu Shang, Xiao-Dan Liu, Da-Fei Xie, Qi Wang, Hua Guan, Ping-Kun Zhou

**Affiliations:** 1 School of Radiation Medicine and Protection, Medical College of Soochow University, Collaborative Innovation Center of Radiation Medicine of Jiangsu Higher Education Institutions, Suzhou, Jiangsu 215123, P R China; 2 Department of Radiation Toxicology and Oncology, Beijing Key Laboratory for Radiobiology, Beijing Institute of Radiation Medicine, Beijing 100850, P R China

## Abstract

Radiation-induced bystander effect (RIBE) describes a set of biological effects in non-targeted cells that receive bystander signals from the irradiated cells. RIBE brings potential hazards to adjacent normal tissues in radiotherapy, and imparts a higher risk than previously thought. Excessive release of some substances from irradiated cells into extracellular microenvironment has a deleterious effect. For example, cytokines and reactive oxygen species have been confirmed to be involved in RIBE process via extracellular medium or gap junctions. However, RIBE-mediating signals and intercellular communication pathways are incompletely characterized. Here, we first identified a set of differentially expressed miRNAs in the exosomes collected from 2 Gy irradiated human bronchial epithelial BEP2D cells, from which miR-7-5p was found to induce autophagy in recipient cells. This exosome-mediated autophagy was significantly attenuated by miR-7-5p inhibitor. Moreover, our data demonstrated that autophagy induced by exosomal miR-7-5p was associated with EGFR/Akt/mTOR signaling pathway. Together, our results support the involvement of secretive exosomes in propagation of RIBE signals to bystander cells. The exosomes-containing miR-7-5p is a crucial mediator of bystander autophagy.

The radiation-induced bystander effects (RIBEs) describes a set of biological effects occurring in the non-targeted cells as a consequence of receiving signals or effective factors from the ionizing radiation (IR)-exposed neighboring cells^1^,^2^. In 1992, Nagasawa and Little first provided the evidence to demonstrate the phenomenon of RIBEs through revealing that the low dose of α-particles induced a more serious biological damage than what was attributable to the dose itself^2^. The RIBEs changed the paradigm of our knowledge in radiobiological effects, and clearly showed that the deleterious effects of IR are not only due to the nuclear DNA damage but also from cytoplasm or extracellular signaling events, i.e. non-target effect^3^. The mechanisms of RIBEs and its significance of health effects are still main topics of radiation oncology, radiobiology and protection.

To date, a great deal of studies proved the existence of RIBEs *in vivo*^4^,^5^ and *in vitro*^6^,^7^. A set of RIBEs endpoints have been reported, including micronuclei^8^,^9^,^10^, gene mutation^11^,^12^,^13^, chromosomal aberration^14^, DNA damage^8^,^15^,^16^,^17^,^18^, apoptosis or cell killing^19^,^20^,^21^, inflammatory response^22^,^23^,^24^, etc. Recently, Wang *et al*. reported that the expression of the autophagy markers LC3-II/LC3-I and Beclin-1 increased in the bystander HepG2 cells treated with conditioned medium (CM) collected from the irradiated HepG2 cells^25^. Transfecting of LC3 siRNA or Beclin-1 siRNA significantly enhanced the yield of micronuclei in bystander cells, suggesting autophagy might also play a role in modulating the bystander effects. Autophagy is a lysosomal degradation pathway in eukaryotic cells activated by variety of stimuli to recycle obsolete cellular components and remove the damaged proteins and organelles. Autophagy is reported to have a cytoprotective role in response to various forms of cellular stresses, including deprivation of nutrients, hypoxia and genotoxic agents, such as ionizing radiation^26^,^27^,^28^. Despite its predominant function as a potential survival mechanism, accumulating data also demonstrated that autophagy represents a pathway contributing to cell death^28^,^29^. The role, and mediating factor(s) and mechanism of autophagy in RIBEs are still not clear.

As reported, there are two major mechanistic pathways of transmitting the signals of RIBEs from irradiated cells to the non-irradiated bystander cells. First, through the gap junction intercellular communication the signals transmit from these directly irradiated cells into the non-irradiated contacted neighboring cells^15^,^30^,^31^. Second, a series of secreted factors such as cytokines^15^,^16^,^32^,^33^,^34^,^35^ or soluble signals such as reactive oxygen species (ROS) and nitric oxide^16^,^17^,^36^,^37^ trigger the RIBEs through the medium communications between the targeted cells and the distanced non-targeted cells. Therefore, the bystander effectors can be transferred through culture medium to the cells situated at a longer distance from the irradiated cells, which has a special significance in consideration of normal tissues injury in cancer radiotherapy. Recently, the exosome, which is a small membrane-bound nanovesicle, has also been reported to deliver the signals from the irradiated cells to the bystander cells through medium transferring^38^,^39^,^40^,^41^.

An exosome is a cell-derived nanovesicle with the size ranging from 30–120 nm, which are originated from endocytic compartments and are released by various types of cells into the extracellular environment^42^. After release, the exosomes are endocytosed by recipient cells and therefore are recognized as an important signal factor to mediate cell-cell communication. The components of exosomes are complex. Except for the constructive lipids and kinds of proteins, several recent studies indicated that exosomes also contain nucleic acids, including DNA, mRNA and small non-coding miRNAs, etc^42^,^43^. miRNAs are a class of endogenous short noncoding RNAs with 19–23 nucleotides which repress translation or degrade target mRNAs by binding to the 3′-untranslated regions (UTRs). miRNAs have significant roles in various types of cellular biological processes through modulating gene expression at the post-transcriptional level. A range of investigations have revealed the involvement of miRNAs in biological response of ionizing radiation^44^,^45^,^46^. Furthermore, recent studies proved that miRNAs, which includes even the miRNAs loading in the exosome, also play important roles in RIBEs. Xu *et al*. found that miR-21 was significantly upregulated in both irradiated or bystander MRC-5 cells, and miR-21 mimics caused bystander-like effects in the non-irradiated cells^47^. More interestingly, the expression profile of miRNA in bystander cells/tissues seems to be distinguished from that of irradiated cells/tissues^45^. Another current work showed that pretreatment of RNase could partially impair the exosomes-mediated RIBEs, suggesting the RNA molecule of exosomes is a novel mediator of RIBEs^48^. However, more details of exosomal miRNA molecules in RIBEs processes need to be clarified.

In this study, we used the human bronchial epithelial cell line BEP2D as the normal cellular model to address the biological effects of radiation-induced exosomal miRNAs. BEP2D cell line is cultured in serum-free medium, which could decrease the interference of serum factor on exosome formation and harvest. We first identified the differentially expressed miRNAs in the exosomes from the culture medium of human bronchial epithelial cells BEP2D irradiated with 2 Gy γ-rays in comparison with the miRNAs in the exosomes from non-irradiated cells. Then the radiation-inducible exosomal miRNAs were tested to determine if they triggered the bystander effect or not, and what kind of bystander endpoints was induced. Our results show that the exosomes from the irradiated cells can induce the autophagy of non-irradiated cells. The exosomal miR-7-5p was confirmed to mediate this bystander autophagy. We also investigated the mechanism through which miR-7-5p induces autophagy in bystander cells.

## Results

### Identification of IR-inducible miRNAs in the exosomes from irradiated cells

**Figure 1:**
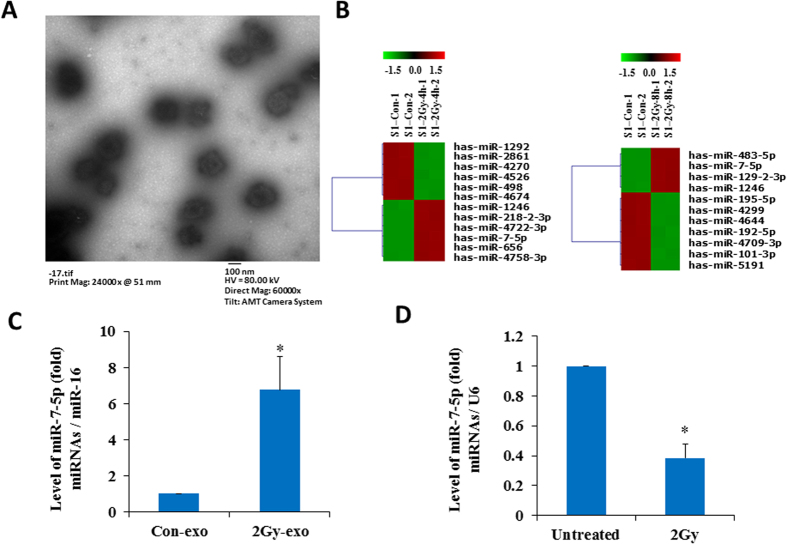
Identification of miRNA profiles in the secretive exosomes from 2 Gy –irradiated BEP2D cells. Panel A: Image of the exosomes under transmission electron microscope (TEM). A number of exosomes particles of 50–120 nm are shown in the image. BEP2D cells were irradiated with 2 Gy of ^60^Co γ-rays, 4 hr later the supernatant exosomes were harvested and identified with TEM. Panel B: Microarray analysis displays the differentially expressed miRNAs in the exosomes from 2 Gy-irradiate BEP2D cells as compared with these from control non-irradiated cells. Panel C: The change of exosomal miR-7-5p expression at 4 h post-2 Gy irradiation was confirmed by RT-qPCR. *p < 0.01 as compared with the exosomes from non-irradiated control cells. Panel D: Intracellular miR-7-5p expression in BEP2D cells at 4 h post-2 Gy irradiation was detected by RT-qPCR. *p < 0.01 as compared with non-irradiated control cells.

The supernatant medium of BEP2D cells cultures were harvested 4 h and 8 h after 2 Gy ^60^Co γ-rays irradiation, and the exosomes were isolated from the medium by the procedure of filtration and super-speed centrifugation. The exosomes pellets were examined by transmission electron microscopy (TEM), a number of 50–120 nm diameter of exosome particles were visible in Fig. 1A. To identify the IR-inducible miRNAs in the exosomes, we have compared the expression profile of miRNAs between the exosomes collected from irradiated cells and non-irradiated control cells by miRNA microarray analysis. Six upregulated miRNAs and six downregulated miRNAs have been identified at 4 hr post 2 Gy irradiation (Fig. 1B left and Supplementary Table 1). At 8 hr post 2 Gy irradiation, four upregulated miRNA and seven downregulated miRNAs were identified (Fig. 1B right and Supplementary Table 2). The changed expression levels of these exosomal miRNAs by IR are all statistically significant (*p* < 0.01). Among them, the increase of miR-7-5p and miR-1246 is sustained from 4 hr to 8 hr after irradiation. The changed levels of other miRNA were found distinctly at 4 hr or 8 hr after irradiation. The IR-induced expression changes of some exosomal miRNAs were further confirmed by RT-qPCR (Supplementary Table 3). Although the level of miRNA-7-5p increased more than five times in exosomes collected from the irradiated cells’ culture medium (Fig. 1C and supplementary table 3), the intracellular miRNA-7-5p levels decreased in the irradiated cells (Fig. 1D). We have performed the biological pathways analysis of the predicted target genes of these exosomal miRNAs based on the KEGG (Kyoto Encyclopedia of Genes and Genomes) database using the Cytoscape software. A set of biological pathways have been displayed in Supplementary Fig. 1, including splicesome pathway, Gap junction, lysosome, ubiquitin-mediated proteolysis, apoptosis pathway, p53 signal pathway, cell cycle pathway, MAPK pathway, etc. However, additional experimental studies are required to further elucidate whether these predicted biological pathways indeed involve in the biological effects of irradiation-related exosomes.

### Induction of autophagy by miR-7-5p mimic

**Figure 2:**
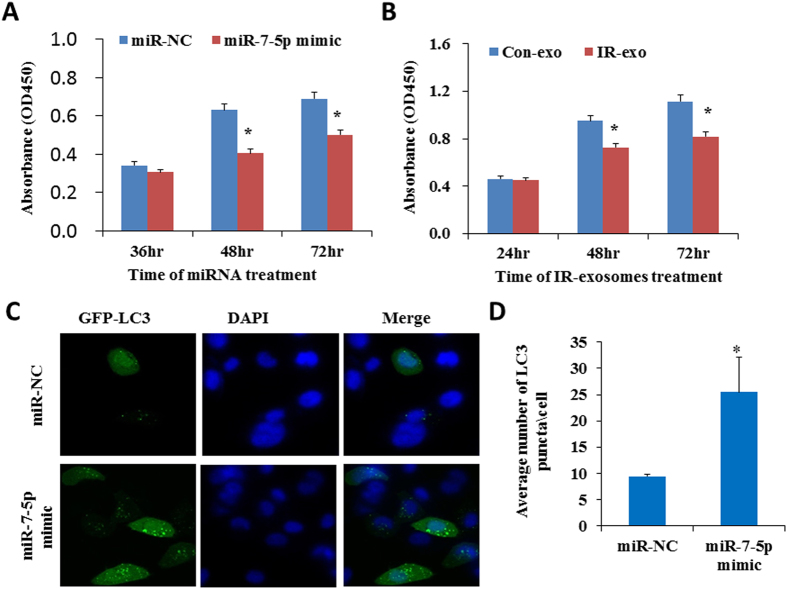
Induction of autophagy by miR-7-5p in BEP2D cells. Panel A: BEP2D cells were transfected with 50 nM of miR-7-5p mimic or miR-NC, cell proliferation was evaluated by CCK-8 assay at 36 hr, 48 hr and 72 hr. *p < 0.01 as compared with control miR-NC treated cells. Panel B: BEP2D cells were treated with the exosomes from 2 Gy-irradiated cells or non-irradiated cells, cell proliferation was evaluated by CCK-8 assay at 36 hr, 48 hr and 72 hr. *p < 0.01 as compared with the cells treated with exosomes from non-irradiated cells. Panel C: BEP2D cells were co-transfected with miR-7-5p mimic or miR-NC and pEGFP-C1-LC3 vector, respectively. 24 hr later, autophagic vacuoles were detected by fluorescent staining. Nuclei were stained blue with DAPI, while green was marker LC3-GFP. Panel D: The number of autophagosomes was counted in 20 randomly selected positive cells. *p < 0.01 as compared with control miR-NC treated cells.

**Figure 3:**
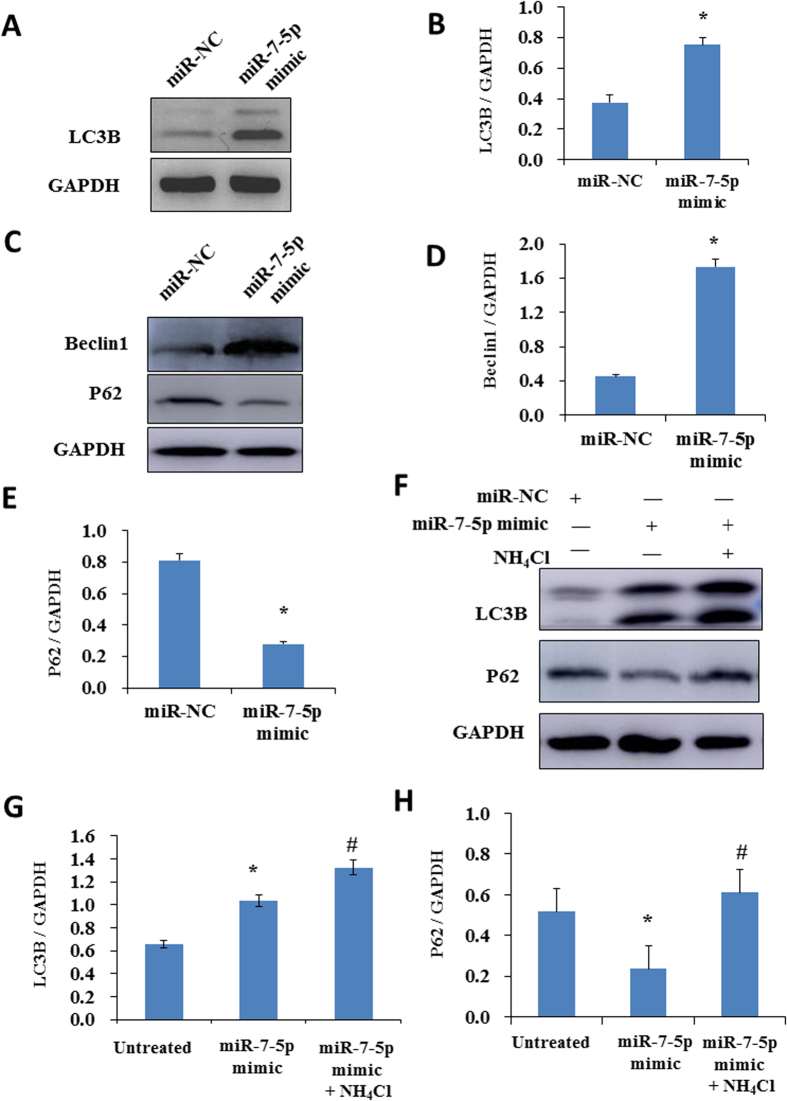
The effects of miR-7-5p on the expression of autophagy related proteins. Panel A: Expression of the autophagy marker LC3B was detected by Western blotting analysis in BEP2D cells 24 hr after transfected with 50 nM of miR-NC or miR-7-5p mimic. Panel B: Densitometric quantitation of western blotting analysis of LC3B protein expression represented in panel A. *p < 0.01 as compared with control miR-NC treated cells. Panel C: Expression of the autophagy markers Beclin 1 and P62 were detected by Western blotting analysis in BEP2D cells 24 hr after transfected with miR-7-5p or miR-NC as above. Panel D: Densitometric quantitation of western blotting analysis of Beclin 1 protein expression represented in panel C. *p < 0.01 as compared with control miR-NC treated cells. Panel E: Densitometric quantitation of western blotting analysis of P62 protein expression represented in panel C. *p < 0.01 as compared with control miR-NC treated cells. Panel F: The analysis of autophagy flux. BEP2D cells were treated with miR-7-5p or miR-NC in the presence or absence of lysosomal inhibitor NH_4_Cl for 24 hr, LC3B and P62 proteins were detected by Western blotting analysis. Panel G: Densitometric quantitation of western blotting analysis of LC3B protein expression represented in panel F. *p < 0.01 as compared with untreated cells; ^#^*p* < 0.05 as compared with miR-7-5p mimic treated cells. Panel H: Densitometric quantitation of western blotting analysis of P62 protein expression represented in panel F. *p < 0.05 as compared with untreated cells; ^#^*p* < 0.05 as compared with miR-7-5p mimic treated cells.

The biological effect of exosomal miRNA-7-5p on bystander cells attracts our attention. It was previously reported that miR-7-5p suppresses cell proliferation and induces apoptosis of cancer cells^49^,^50^. We have first investigated the effect of miR-7-5p as well as the exosomes from the irradiated cells on BEP2D cells proliferation activity using CCK-8 assay. The results indicated that the treatment of miR-7-5p mimics (Fig. 2A) or the exosomes from irradiated cells (IR-exo) (Fig. 2B) obviously suppressed the proliferation of BEP2D cells. However, our results showed that there was not significant difference on the proportion of apoptosis (Supplementary Fig. 2A,B) and cell cycle distribution (Supplementary Fig. 2C) between miR-7-5p mimics treated BEP2D cells and control cells. We have further investigated whether miR-7-5p induces autophagy of BEP2D cells or not. miR-7-5p mimic or the control miR-NC were co-transfected with GFP-LC3 fusion vector into BEP2D cells, repectively. The autophagy was determined by monitoring the number of GFP-LC3 puncta in the cells after co-transfection. As shown in Fig. 2C,D, the number of GFP-LC3 puncta were significanlly increased in miR-7-5p mimics transfected BEP2D cells in compare with the negative control cells. In addition to the formation of GFP-LC3 puncta, conversion of LC3 from the cytosolic (LC3-I) to the lipid-bound form (LC3-II) were also detected by western blot analysis, the yield of LC3-II was greatly increased in miR-7-5p mimics transfected BEP2D cells as compared with miR-NC treated BEP2D cells (Fig. 3A,B). In addiation, we have also observed by western blot analysis that Beclin 1 protein levels increased (Fig. 3C,D) and the levels P62 protein decreased (Fig. 3C,E). In order to clarify whether the increase in LC3B was due to an enhancement of vesicles formation or an impairment of vesicle fusion with the lysosome, the autophagy flux was investigated. As shown in Fig. 3F,G, in the presence of lysosomal inhibitor NH_4_Cl, LC3B protein levels further increased whereas the decrease in P62 content was attenuated (Fig. 3F,H). Therefore, the accumulation of LC3B after miR-7-5p treatment was due to the enhanced formation of vesicles.

### miR-7-5p mediates the autophagy induction by the exosomes from irradiated cells

**Figure 4:**
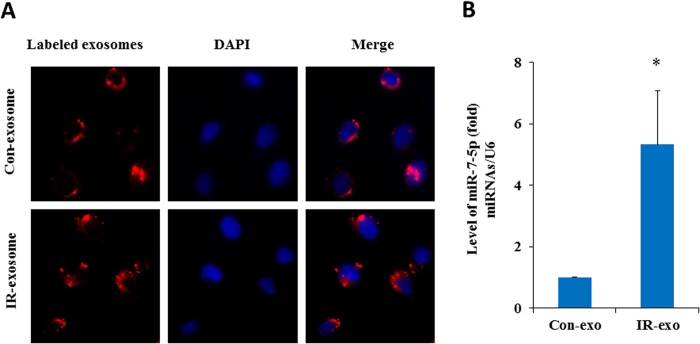
Exogenous exosomes uptake and detection of miR-7-5p in the recipient cells. Panel A: BEP2D cells were irradiated with or without 2 Gy of ^60^Co γ-rays, 4 hr later, the exosomes were collected from the medium of cells cultures. The exosomes were labeled with CM-Dil fluorescent dye, and then added to the culture of non-irradiated BEP2D cells. The exosomes uptake was observed by the fluorescent staining. Nuclei were stained blue with DAPI, while exosomes were stained red. Panel B: RT-qPCR estimated the level of miR-7-5p in the non-irradiated cells upon uptake of exogenous exosomes from irradiated cells (IR-exo) or non-irradiated cells (Con-exo). *p < 0.01 as compared with the cells treated with the control exosomes (con-exo) from non-irradiated cells.

To determine whether the exosomes secreted by the IR-irradiated cells can shuttle into the bystander cells and cause bystander effects, we labeled the exosomes from the conditioned medium of 2 Gy irradiated cells with CM-Dil fluorescent dye. As visible by fluorescence microscopy, the recipient BEP2D cells exhibited high uptake efficiency of the exosomes from the IR-irradiated cells (IR-exosome) as well as non-irradiated cells (Con-exosome) (Fig. 4A). Moreover, a dramatically increased level of miR-7-5p was detected in recipient cells after intake of the exogenous exosomes from the irradiated cells (IR-exo) as compared to that from the non-irradiated cells (Con-exo) (Fig. 4B). These data indicated that the exosomes mediated the transfer of miR-7-5p from the irradiated cells to the bystander cells where miR-7-5p mediates the non-target effects.

**Figure 5:**
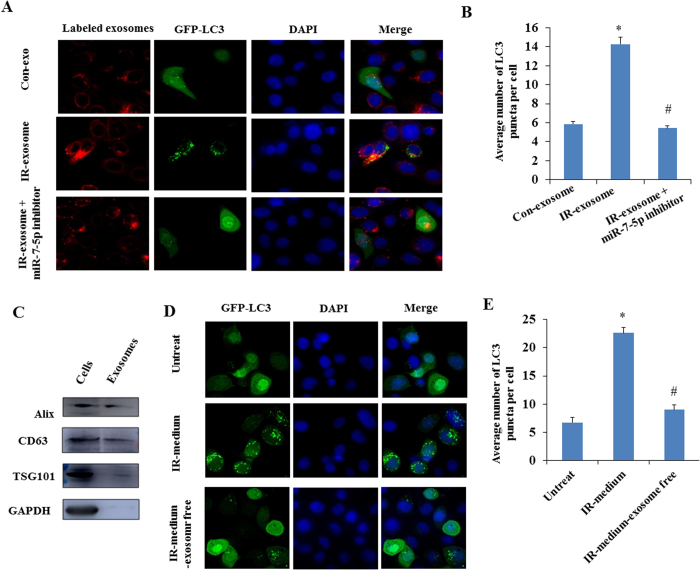
Bystander autophagy induced by the exosomal miR-7-5p in recipient BEP2D cells. Panel A: Observation of autophagy induced by exosomes. BEP2D cells were irradiated with 2 Gy of ^60^Co γ-rays. The exosomes were harvested 4 hr post-irradiation, and co-treated the non-irradiated cells with pEGFP-C1-LC3 vector in the presence (IR-exosome + miR-7-5p inhibitor) or absence (IR-exosome) of miR-7-5p inhibitor. The autophagic vacuoles were determined by fluorescent staining. Nuclei were stained blue by DAPI, while exosomes were stained red, and green was LC3-GFP. The effect of exosomes from the non-irradiated cells were also performed as the control (Con-exosome). Panel B: The number of autophagosomes (LC3 punctium) in the exosomes-treated BEP2D cells was counted in 20 randomly selected positive cells (red and green). *p < 0.01: IR-exosome *vs* Con-exosome. ^#^*p* < 0.01: IR-exosome + miR-7-5p inhibitor *vs* IR-exosome. Panel C: Western blotting analysis of the exosomal proteins Tsg101, Alix, CD63 in BEP2D cells and the exosomes. Panel D: Observation of autophagy induced by the conditional medium from irradiated cells. BEP2D cells were irradiated with 2 Gy of ^60^Co γ-rays. The conditional medium was collected 4 hr post-irradiation. After removing cellular debris by centrifugation, the exosomes-containing conditional medium (IR-medium) and exosome-free medium (IR-medium-exosome free) were used to treat the non-irradiated BEP2D cells. The exosomes-free medium was prepared by further super-speed centrifuging the conditional medium to remove the exosomes at 100,000 g for 70 min. Panel E: The number of autophagosomes (LC3 punctium) in the medium-treated BEP2D cells was counted in 20 randomly selected positive cells (green). *p < 0.01 as compared with untreated cells. ^#^*p* < 0.01 as compared with the cells treated with the medium from irradiated cells.

We next investigated whether the exosomal miR-7-5p induced autophagy in the recipient bystander cells. BEP2D cells were irradiated with 2 Gy of γ-rays. Four hours later, the exosomes were harvested from the medium of 2 Gy-irradiated cells or the non-irradiated control cells, and then added to the cultures of the non-irradiated BEP2D cells together with or without miR-7-5p inhibitor. The autophagy was monitored in the recipient cells by detecting the yield of LC3 punctium image. The result demonstrated that the exosomes from the irradiated cells obviously resulted in autophagy in recipient bystander cells (Fig. 5A,B). Importantly, in the presence of miR-7-5p inhibitor, this bystander autophagy induced by the exosomes from irradiated cells has been significantly suppressed. Figure 5C showed that the exosomes markers, including Alix, TSG101 and CD63, were detected in the exosomes.

The effect of autophay induction has been further investigated for the conditional medium from irradiated cells. As shown in Fig. 5D,E, the conditional medium from 2 Gy-irradiated BEP2D cells (IR-medium) obviously resulted in autophay in the non-irradiated cells. However, this effect of autophagy induction was largely attenuated when the exosomes were removed from the conditional medium by super-speed centrifugation. Simultaneously, we have also observed that the cells growth was slowed down by the conditional medium as compared to the exosome-free conditional medium (Supplementary Fig. 3).

### miR-7-5p induces autophagy through regulating the EGFR signal pathway

**Figure 6:**
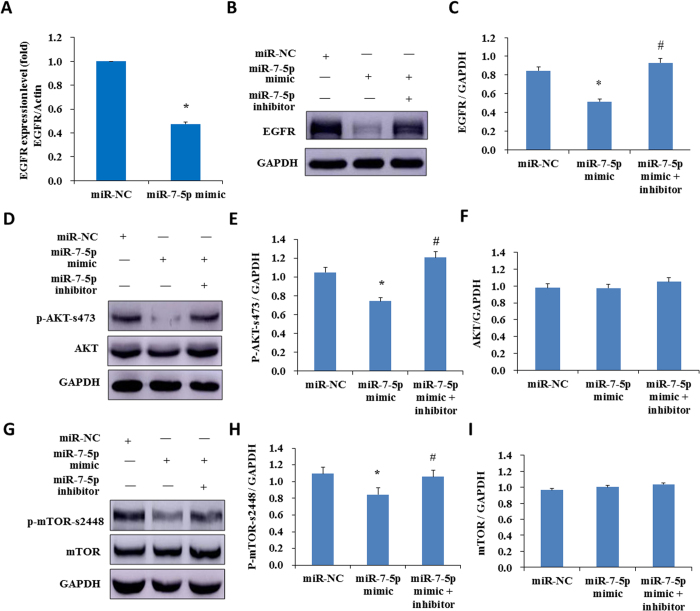
Identification of EGFR signaling as the downstream targets of miR-7-5p. Panel A: BEP2D cells were transfected with miR-7-5p mimic or miR-NC, 24 hr later EGFR mRNA expression was determined by RT-qPCR. *p < 0.01 as compared with the cells transfected with miR-NC. Panel B: EGFR protein level was detected by western blotting analysis in BEP2D cells transfected with miR-7-5p mimic, or miR-7-5p mimic plus miR-7-5p inhibitor, or miR-NC for 24 hr. Panel C: Densitometric quantitation of western blotting analysis of EGFR protein expression represented in panel B. *p < 0.01 as compared with the cells treated with miR-NC. ^#^*p* < 0.01 as compared with the cells treated with miR-7-5p mimic. Panel D: AKT and p-AKT protein levels were detected by western blotting analysis in BEP2D cells transfected with miR-7-5p mimic, or miR-7-5p mimic plus miR-7-5p inhibitor, or miR-NC for 24 hr. Panel E: Densitometric quantitation of western blotting analysis of p-AKT protein expression represented in panel D. *p < 0.05 as compared with the cells treated with miR-NC. ^#^*p* < 0.05 as compared with the cells treated with miR-7-5p mimic. Panel F: Densitometric quantitation of western blotting analysis of AKT protein expression represented in panel D. There is no statistical significance (*p* > 0.05) between miR-NC and miR-7-5p mimic or between miR-7-5p mimic and miR-7-5p mimic + inhibitor. Panel G: mTOR and p-mTOR protein levels were detected by western blotting analysis in BEP2D cells transfected with miR-7-5p mimic, or miR-7-5p mimic plus miR-7-5p inhibitor or miR-NC for 24 hr. Panel H: Densitometric quantitation of western blotting analysis of p-mTOR protein expression represented in panel G. *p < 0.05 as compared with the cells treated with miR-NC. ^#^*p* < 0.05 as compared with the cells treated with miR-7-5p mimic Panel I: Densitometric quantitation of western blotting analysis of mTOR protein expression represented in panel G. There is no statistical significance (*p* > 0.05) between miR-NC and miR-7-5p mimic or between miR-7-5p mimic and miR-7-5p mimic + inhibitor. p-Akt represents phosphorylated protein kinase B, p-mTOR represents phosphorylated mammalian target of rapamycin.

The bioinformatic analysis suggests that EGFR is a potential target of miR-7-5p. Tazawa *et al*. showed that a genetically engineered oncolytic adenovirus induced autophagic cell death via regulating E2F1-miR-7-EGFR axis in human cancer cells^51^. To determined whether EGFR signal pathway also involves in miR-7-5p mediated autophagy in BEP2D cells, the effect of miR-7-5p on EGFR expression was investigated. miR-7-5p mimics or miR-NC were transfected into BEP2D cells and the expression level of EGFR were assessed by western blot and RT-qPCR. The results indicated that both mRNA (Fig. 6A) and protein level of EGFR (Fig. 6B,C) significantlly decreased in miR-7-5p mimics transfected BEP2D cells in compared with control cells. The decreased level of EGFR was largely attenuated by miR-7-5p inhibitor (Fig. 6B,C). Consistent with this, the level of EGFR was also partially decreased in BEP2D cells treated with the exosomes from 2 Gy irradiated BEP2D cells, and which could also be rescued by miR-7-5p inhibitor (Supplementary Fig. 4A,B).

The Akt-mTOR signal pathway localizes at the downstream of EGFR and plays an essential role in regulating of autophagy progression. We have further proved that the phosporylation level of phosphor-Akt and phosphor-mTOR, both of which participate in autophagy regulation, notably decreased when the BEP2D cells were transfected with miR-7-5p mimics (Fig. 6D,E for phosphor-AKT-s473, Fig. 6G,H for phosphor-mTOR-s2448). miR-7-5p inhibitor attenuated the phosphorylation inhibition of Akt and mTOR by miR-7-5p. Our results suggest that the exosomal miR-7-5p induces autophagy in BEP2D cells associating with the EGFR/Akt/mTOR signaling axis.

## Discussion

Ionizing radiation is well known as a significant risk for both acute detriments and long-term carcinogenic events. On the other hand, radiotherapy is an effective measure for cancer therapy. Considering the important role in the radiobiological context, the significance and potential implications of RIBEs for radiotherapy and cancer risk limitation have gained lots of attention in recent years. Despite increasing evidence and emergence of new models, the detailed molecular mechanism of RIBEs still remains to be elucidated. Directly irradiated cells can generate some bystander signaling factors which will be taken up by bystander cells and cause various biological effects in adjacent or physically separated cells. Recent studies have uncovered that exosomes could be another form of signal mediator transmitting RIBEs^38^,^39^,^40^,^41^. In present study, we have revealed that the exosomal miR-7-5p from the irradiated cells mediated a bystander autophagy in the non-irradiated human bronchial epithelial BEP2D cells. Emerging evidence supports that the miRNA can be packaged into exosomes, through which entering into the neighboring or distant cells with circulating in body fluids^52^, and these miRNAs were protected in exosomes to escape from degradation^53^. In this study, the profiles of miRNAs in the secreted exosomes from 2 Gy-irradiated BEP2D cells were identified as compared with exosomes from non-irradiated cells. Our data demonstrated that a set of exosomal miRNAs in the exosomes were significantly upregulated or downregulated by ^60^Co γ-rays irradiation. Among these miRNAs, miR-7-5p level was persistently upregulated at 4 hr and 8 hr post-irradiation. Interestingly, the expression level of miR-7-5p has different change trend in secreted exosomes and intracellular after IR treatment (Fig. 1C,D). We assumed that the downregulation of intracellular miR-7-5p in irradiated BEP2D cells might be due to the package of miR-7-5p into exosomal cargo after irradiation.

We paid special attention to miR-7-5p after further validation of its increased expression in the exosomes from irradiated cells with RT-qPCR analysis. Our data revealed that exosomal miR-7-5p induced autophagy in the non-targeted bystander cells. Autophagy is a lysosomal degradation pathway in eukaryotic cells activated by variety of chemical and physical stresses such as ionizing radiation. There are plenty of reports demonstrating that autophagy leads to dual effects of protecting cells survival and promoting cells death under different cellular condition^28^,^29^. Recently, Wang *et al*. demonstrated that the expression of LC3-II/LC3-I and Beclin-1, which are autophagy markers, increased in IR bystander human hepatoma cells, and the inhibition of autophagy significantly enhanced the presence of micronuclei (MN) in bystander cells^25^. Our data further supports that the autophagy represents another key endpoint of RIBEs. Considering the fact that both miR-7-5p mimic and exosomes from the irradiated cells suppressed the cells proliferation, we deemed that the autophagy induced by the exosomal miR-7-5p might play the role of producing a detrimental effect.

Although previous report has shown that the exosomal RNA from the irradiated cells induced the bystander effects of DNA/chromosomal damage^38^, and our bioinformatics analysis also implied that apoptosis pathway and cell cycle pathway were included in the biological pathways of the predicted targets genes of the IR-induced exosomal miRNAs, miR-7-5p was not shown the effects of inducing DNA damage, cell cycle change and apoptosis in present study. Therefore, if the IR-induced exosomes could activate the apoptosis pathway or regulate cell cycle, it might attribute to other miRNAs or other signal molecules in the exosomes. We have further determined that the exogenous exosomes from the irradiated cells could be taken up by BEP2D cells, and also induced the autophagy in the non-targeted cells. Our data demonstrated that the bystander autophagy induced by exosomes could be effectively attenuated by miR-7-5p inhibitor. As both miR-7-5p mimic and exosomes from the irradiated cells suppressed the cells proliferation, suggesting that the autophagy induced by the exosomal miR-7-5p might result in a detrimental effect of RIBEs.

A recent paper has demonstrated that EGFR is a direct target of miR-7-5p^54^. EGFR is an oncogenic receptor tyrosine kinase, and has a broad of biological functions implicating in cellular proliferation, metastasis, angiogenesis, and chemo/radioresistance of cancer cells. A series reports have demonstrated that EGFR is a key factor involving in autophagy regulation, suppression of EGFR induced autophagy in multiple cancer cell lines^55^,^56^,^57^,^58^. As the downstream targets of EGFR signaling, Akt/mTOR axis also plays an essential role in autophagy^55^,^59^. Our results indicated that miR-7-5p suppressed the expression of EGFR, and consequently the phosphorylation of Akt and mTOR was also depressed. The exosomes from irradiated cells also led to the suppression of EGFR in the recipient cells, and which could be rescued by miR-7-5p inhibitor. Therefore, we suggest that the exosomes mediate miR-7-5p transfer to the non-target cells, where miR-7-5p induces autophagy through targeting EGFR signaling pathway.

In summary, ionizing radiation may prompt the package of a set of miRNAs into exosomes, which mediate the delivery of these molecules into the recipient cells. As one of IR-upregulated exosomal miRNAs, miR-7-5p can induce autophagy in the non-irradiated cells through targeting EGFR/Akt/mTOR signaling pathway. Our results have provided further evidence to support that exosome is an effective mediator of RIBEs. It is valuable to pay more attention to the mechanism how ionizing radiation influences the choice and package of some special exosomal molecules such as miR-7-5p, and which may play a role in the processing of bystander effects in non-targeted cells. Our investigations provided advanced acknowledge which could be valuable for designing a better strategy of diminishing normal tissue injury in cancer radiotherapy.

## Materials and Methods

### Cell culture and irradiation

Human bronchial epithelial cell line (BEP2D) cells were obtained from Dr CC Harris (Laboratory of Human Carcinogenesis Division of Basic Science, National Cancer Institute, NIH, USA). The cells were maintained in serum-free LHC-8 medium (Gibco, USA) supplemented with 100 units per ml of penicillin and 100 μg/ml of gentamycin in a humidified incubator at 37 °C with 5% CO_2_. Cells were irradiated with ^60^Co γ-rays at a dose rate of 1.98 Gy/min at room temperature.

### Exosomes isolation and miRNA microarray analysis

The BEP2D cells were seeded in the 10 cm diameter of dishes (Thermo, China). After 24 hours incubation, 10 ml of fresh serum LHC-8 medium were replaced and the cells were irradiated by 2 Gy of ^60^Co γ-rays. The cells were further cultured for 4 hr and 8 hr, and the control cells were established in parallel. The conditional medium (CM) from the irradiated BEP2D cells and control medium from non-irradiated control cells were collected and filtered with 0.2 μm filters (PALL,USA), respectively. The medium was first centrifuged at 300 g for 10 minutes and at 2000 g for 20 minutes at 4 °C to remove cells debris. The supernatants were then centrifuged at 100,000 g for 70 minutes at 4 °C. The pellets of exosomes were re-suspended in 100–200 μl of sterile 1× phosphate buffered saline. Exosomes RNAs were extracted using TRIzol (Sigma, USA). The exosomes miRNA microarray analysis was performed by LC Sciences (Houston, Texas).

### Transmission electron microscopy observation

The exosomes obtained after super-speed centrifugation were fixed in 2% phosphate-tungstic acid. A 10-μl drop of the suspension was loaded onto Formvar-carbon-coated electron microscopy copper grids, excess fluid was drawn off with a piece of Whatman filter paper. The sample was then negative-stained with 2% phosphato-tungstic acid at pH 6.8 stained for 1 minute, again drawn off with a piece of Whatman filter paper, and allowed to dry under an electric incandescent lamp for 10 minutes before viewing with the transmission electron microscope Hitachi H-7650 (Japan) operated at 80 kv.

### Exosome labeling and analysis

The re-suspended exosomes were stained with the fluorescent dye CM-Dil, 3H-Indolium, 5-[[[4-(chloromethyl) benzoyl]amino]methyl]-2-[3-(1,3-dihydro-3,3-dimethyl-1-octadecyl-2H-indol-2-ylidene)-1-propenyl]-3,3-dimethyl-1-octadecyl-,chloride(Invitrogen, USA). The plasma membrane labelling was performed according to the manufacture protocol. Briefly, the CM –Dil reagent was dissolved in DMSO to prepare a stock solution of 1 mM and diluted vybrant CM-Dil solution directly with phosphate-buffered saline (PBS) into 1 μM working solution. 1 ml of re-suspended exosomes was stained with 1–5 μl of working solution. The exosomes in the working solution were incubated for 10 minutes or less at 37 °C. After labeling, exosomes were washed with PBS and centrifuged at 100000 g for 70 minutes at 4 °C. The exosomes were re-suspended in 100–200 μl of PBS.

### Plasmid, miR-7-5p mimic/inhibitor

pEGFP-C1-LC3 vector was generated by polymerase chain reaction (PCR) cloning technology. miR-7-5p mimic (sense:5′-UGGAAGACUAGUGAUUUUGUUGU-3′, antisense: 5′-AACAAAAUCACUAGUCUUCCAUU-3′), miR-7-5p inhibitor (5′-ACAACAAAAUCACUAGUCUUCCA-3′), and miR-NC (control miRNA) (sense: 5′UUCUCCGAACGUGUCACGUTT) were purchased from GenePharma (Shanghai, China).

### Flow Cytometry for Cell Cycle and Apoptosis Detection

BPE2D cells were transfected with 50 nM of miR-7-5p mimic or miR-NC, 24 h later, the transfected cells were collected and washed with PBS once and re-suspended with 75% ethyl alcohol for fixing more than 12 hr at 4 °C. These samples were washed with PBS, digested with 50 μg/ml of RNase A for 30 min at 37 °C, and then stained with propidium iodide (PI) of 50 μg/ml for 15 min at room temperature. The cells were analyzed using flow cytometry. Each experiment was performed in triplicate. For apoptosis detection, BPE2D cells were transfected with 50 nM of miR-7-5p mimic or miR-NC, 24 hr later, the transfected cells were collected and stained with Alexa Fluor 488 Annexin V and PI for 5 minutes at room temperature in the dark according to the protocol provided by the manufacturer of Apoptosis Detection Kit (DOJINDO, Japan). After staining, the number of cells undergoing apoptosis was determined by flow cytometry analysis. Each experiment was performed in triplicate.

### Cell Proliferation Assay

Cell proliferation assay was performed by Cell Counting Kit-8 (CCK-8) colorimetric assay, which determines the number of viable cells. In this assays, the WST-8 agent, 2-(2-methoxy-4-nitrophenyl)-3-(4-nitrophenyl)-5-(2,4-disulfophenyl)- 2H-tetrazolium, monosodium salt, is reduced by dehydrogenases in living cells to give an orange colored product (formazan), which is soluble in the culture medium. The amount of the formazan dye generated by dehydrogenases in cells is directly proportional to the number of living cells. BPE2D cells were transfected with 50 nM of miR-7-5p mimic or miR-NC, 24 h later, the transfected cells were planted in 96-well plates at a density of 5 × 10^3^ cells/well. Each sample was performed in three parallel. Cells viability was determined at 36 hr, 48 hr and 72 hr later using the Cell Counting Kit-8(DOJINDO, Japan). OD value for each well was read on a microplate reader using the Multiskan GO microplate reader (Thermo,USA) at 450 nm to determine cell viability. Each experiment was performed in triplicate.

### RNA Extraction and Real-Time Quantitative PCR (RT–qPCR)

Total RNAs were extracted from BEP2D cells or the exosomes collected from the irradiated or non-irradiated BPE2D cells using TRlzol reagent (Invitrogen) as recommended by the manufacturer. 0.5 μg~1 μg of total RNA was used to reverse transcribe cDNA. Roche TaqMan microRNA expression assay was used to quantitate mature miR-7-5p expression following the manufacturer’s protocol. U6 or miR-16 expression was used as an internal control for miR-7-5p expression. RT-qPCR was performed with a Bio-Rad iCycler & iQ Real-time PCR systems (Bio-Rad) and a fluorescence-labeled FAM RocheTaqMan kit (Hao Qin Biotech (Shanghai) Co. LTD). Each sample was tested three times, and the expression of miR-7-5p in the untreated control was set as to generate the relative expression level in the treated cells.

BPE2D cells were harvested 24 h after transfecting miR-7-5p mimic or miR-NC, and total RNA was extracted using TRlzol reagent. 1 μg of total RNA was used to reverse transcribe cDNA by ReverTra Ace. RT-qPCR was performed for detecting EGFR expression with a Bio-Rad iCycler & iQ Real-time PCR systems (Bio-Rad) and a fluorescence-labeled SYBR Green real master Mix kit (TIANGEN Biotech (Beijing) Co. LTD). Actin was used as an endogenous control. The sequence of forward and reverse primers for EGFR and actin are the following: EGFR forward 5′-AGGCACGAGTAACAAGCTCAC-3′and reverse 5′-ATGAGGACATAACCAGCCACC-3′; actin forward 5′-CAGAGCAAGAGAGGCATCCT-3′ and reverse 5′-TTGAAGGTCTCAAACATGAT-3′. Each sample was tested three times, and the expression of EGFR in the miR-NC group was set as to generate the relative expression level in the treated cells.

### Antibodies and immunoblotting

All antibodies were commercial products, some primary antibodies were purchased from Santa Cruz Biotechnology, such as p62 (H-12,sc-55604) at a 1:1000 dilutions, Alix (1A12, sc-53540) at a 1:500 dilutions, CD63(H-193, sc-15363) at a 1:500 dilutions, LC3B(G-9, sc-376404) at a 1:500 dilutions, and EGFR (1005, sc-03) at a 1:1000 dilutions. Other primary antibodies were purchased from Cell Signaling Technology, such as Beclin1 (2A4,#4122) at a 1:1000 dilutions, mTOR (#2972S) at a 1:1000 dilutions, p-mTOR-s2448 (#5536S) at a 1:1000 dilutions and p-AKT-s473 (#9271) at a 1:1000 dilutions. The primary antibody against AKT (ab124341) at a 1:1000 dilutions was purchased from Abcam Technology. The primary antibody against GAPDH(TA-08,#15900409) at a 1:1000 dilutions was purchased from Zhong Shan Jin Qiao. For the immunoblotting (western blotting) analyses, BEP2D cells were transfected with miR-7-5p mimic, miR-7-5p inhibitor or miR-NC, 24 hr later, the total proteins were isolated. 30–50 μg protein from each sample were loaded onto SDS-PAGE, then separated and transferred onto a Nitrocellulose membrane (Millipore,USA). The blotting membranes were blocked with 5% milk power in TBST (20 mM Tris-HCl, 500 mM NaCl (pH7.5), 0.1% (v/v) Tween 20) for 1 hr at room temperature, incubated overnight at 4 °C. Membranes were then incubated with the appropriate secondary antibody linked to horseradish peroxidase at a 1:4000 dilution for 1 hr at room temperature, and washed it with TBST. Bands were visualized by Image quant LAS500 (GE).

### Microscopy imaging of fluorescent-staining

For fluorescent-staining, BEP2D cells were co-transfected with 1 μg of pEGFP-C1-LC3 (green fluorescent), and 50 nM of miR-7-5p mimic, miR-7-5p inhibitor or miR-NC for 24 hr, then fixed in chilled phosphate-buffer saline (PBS) containing 4% paraformaldehyde overnight at 4 °C. The fixed cells were permeated in PBS containing 0.25% Trion X-100 for 15 minutes and washed with PBS for 3 times at room temperature, stained with DAP1(Sigma) to visualize the DNA at room temperature 20 minutes away from light, washed with PBS for 3 times again, and observed using a LSM 510 laser scanning confocal microscope (Zeiss,Germany). The autophagic vacuole were visible, three independent experiments were performed.

### Statistical analysis

The Student’s t-test was used for the comparison between two groups and the Bonferroni’s post-hoc test was used to evaluate multiple groups, the statistically significance of the results, *p* < 0.05 was considered significant.

## Additional Information

**How to cite this article**: Song, M. *et al*. Bystander autophagy mediated by radiation-inducible exosomal miR-7-5p in non-targeted human bronchial epithelial cells. *Sci. Rep.* **6**, 30165; doi: 10.1038/srep30165 (2016).

## Supplementary Material

Supplementary Information
