# Supplementary material for: Bystander autophagy mediated by radiation-induced exosomal miR-7-5p in non-targeted human bronchial epithelial cells
Source: Sci Rep. 2016 Jul 15;6:30165. doi: 10.1038/srep30165 (PMC4945935; doi:10.1038/srep30165)
Supplement: Supplementary Information [file srep30165-s1.pdf]

Bystander autophagy mediated by radiation-induced exosomal miR-7-5p in non-targeted human bronchial epithelial cells

Man Song[1,2], Yu Wang[2], Zeng-Fu Shang[1], Xiao-Dan Liu[2], Da-Fei Xie[2], Qi Wang[2], Hua Guan[2*], Ping-Kun Zhou[1,2*]

[1]School of Radiation Medicine and Protection, Medical College of Soochow University, Collaborative Innovation Center of Radiation Medicine of Jiangsu Higher Education Institutions, Suzhou, Jiangsu 215123, P R China
[2]Department of Radiation Toxicology and Oncology, Beijing Key Laboratory for Radiobiology, Beijing Institute of Radiation Medicine, Beijing 100850, P R China

[*] Correspondence and requests for materials should be addressed to H.G (ghlsh@163.com), P.K.Z. (zhoupk@bmi.ac.cn)

Supplementary table 1. Exosomal miRNA expression changes at 4 h post 2 Gy irradiation

	**miRNA ID**	**Change fold /Log2**	***p* - value**
**Upregulated**	hsa-miR-7-5p	2.07448216	7.68E-03
	hsa-miR-1246	2.00762922	7.71E-03
	hsa-miR-4722-3p	12.5945426	3.31E-04
	hsa-miR-656	11.3717221	4.20E-03
	hsa-miR-218-2-3p	11.6770873	7.51E-03
	hsa-miR-4758-3p	2.47257861	9.40E-03
**Downregulated**	hsa-miR-498	-3.0047916	2.23E-03
	hsa-miR-4674	-2.2492524	5.44E-03
	hsa-miR-2861	-1.065818	9.06E-03
	hsa-miR-4526	-1.996437	9.49E-03
	hsa-miR-4270	-1.3081067	3.95E-03
	hsa-miR-1292	-3.2374553	9.31E-03

Supplementary table 2. Exosomal miRNA expression changes 8 h post 2 Gy irradiation

	**miRNA ID**	**Change fold / Log2**	***p* - value**
**Upregulated**	hsa-miR-483-5p	0.27270384	1.67E-03
	hsa-miR-7-5p	3.17076104	6.46E-03
	hsa-miR-1246	3.05500464	6.63E-03
	hsa-miR-129-2-3p	3.18420171	7.87E-03
**Downregulated**	hsa-miR-101-3p	-1.302908	6.20E-03
	hsa-miR-192-5p	-2.4677757	8.67E-03
	hsa-miR-4709-3p	-1.6066929	3.07E-03
	hsa-miR-4299	-2.8222534	6.06E-03
	hsa-miR-195-5p	-2.1918335	6.21E-03
	hsa-miR-5191	-12.128679	6.70E-03
	hsa-miR-4644	-1.6284682	8.33E-03

Supplementary table 3. Validation of IR-induced differential expression of exosomal miRNAs by RT-qPCR (The samples were collected at 4 hr post 2 Gy irradiation)

No	Test miRNA Name	Non-IR Nominal CT Mean	2 Gy-IR Nominal CT Mean	ΔΔCt	Fold Change	Up/ Down	p-value	Control miRNA Name
1	hsa-miR-100-5p	25.60	23.08	2.52	5.72	Up	2.26E-04	hsa-miR-16
2	hsa-miR-1246	22.36	20.04	2.31	4.97	Up	1.84E-04	hsa-miR-16
3	hsa-miR-29b-3p	24.57	23.78	0.79	1.73	Up	1.56E-03	hsa-miR-16
4	hsa-miR-7-5p	21.63	19.56	2.07	4.19	Up	1.32E-03	hsa-miR-16

Supplementary Figure 1

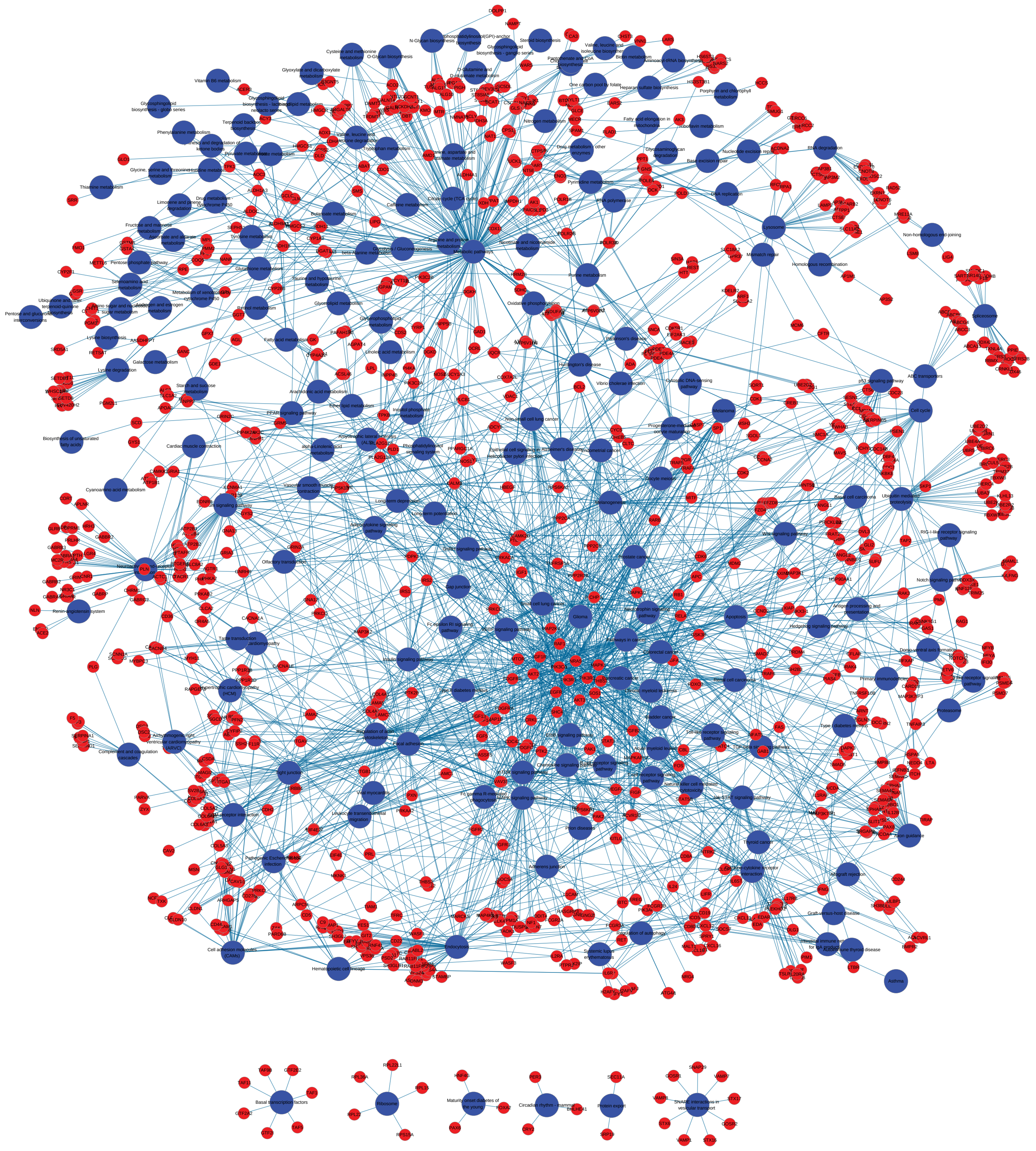

Supplementary Figure 2

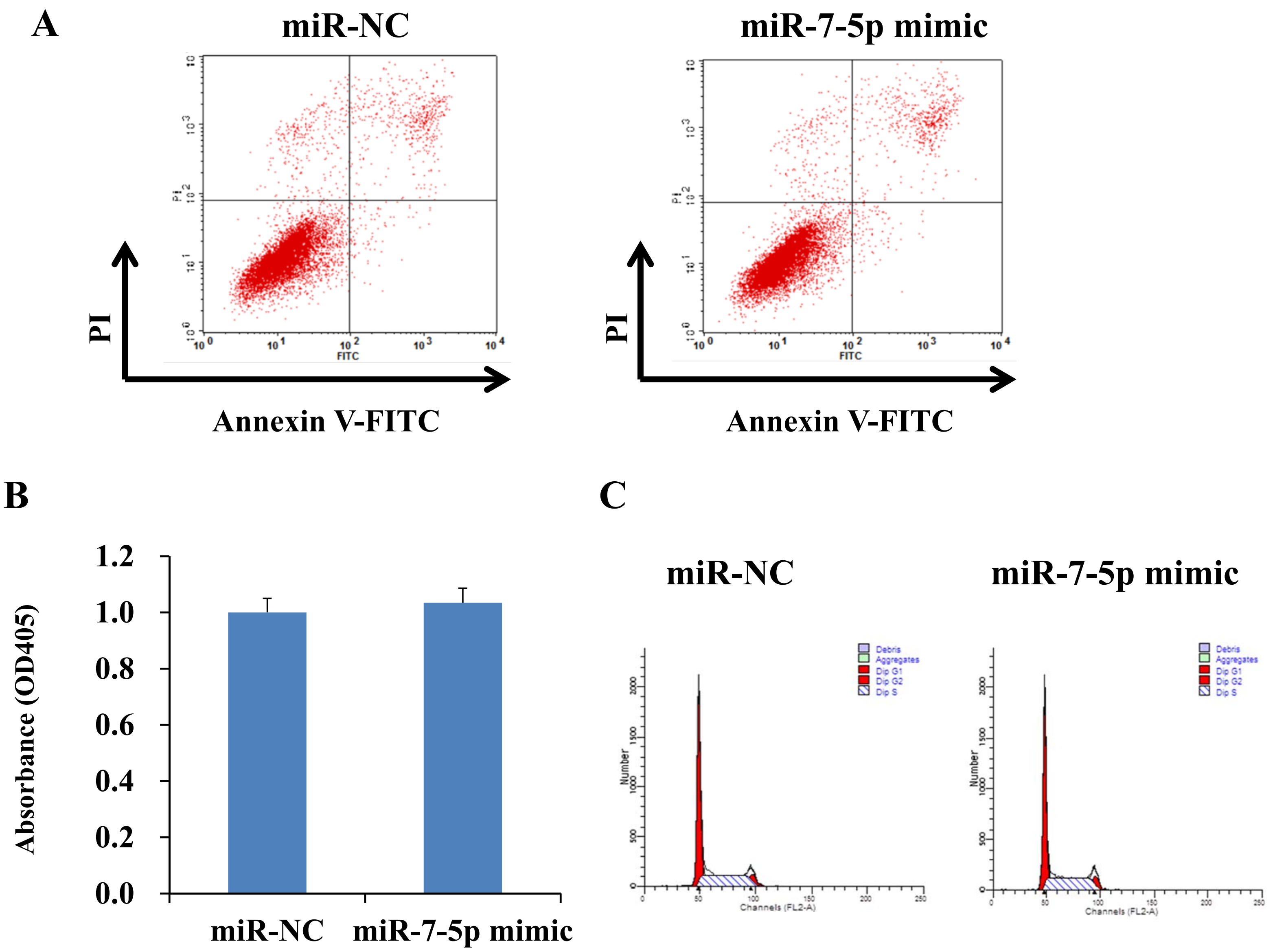

Supplementary Figure 3

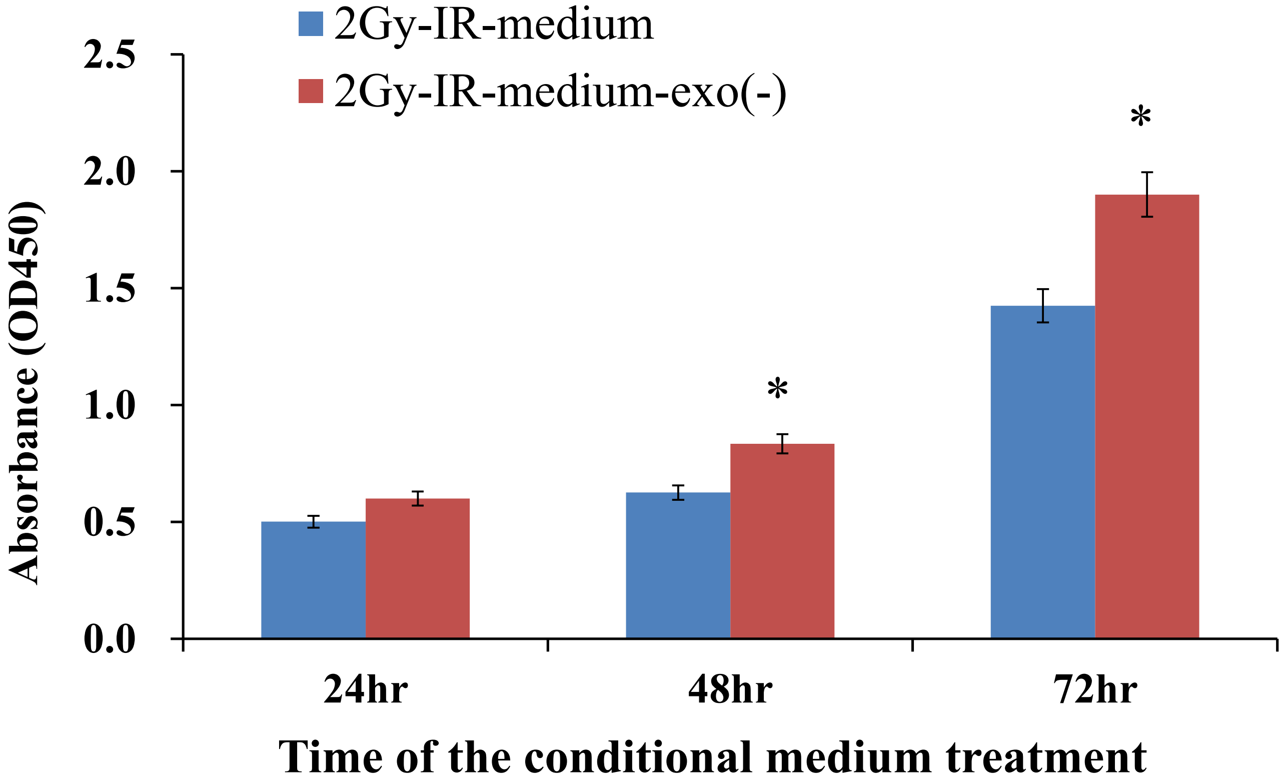

Supplementary Figure 4

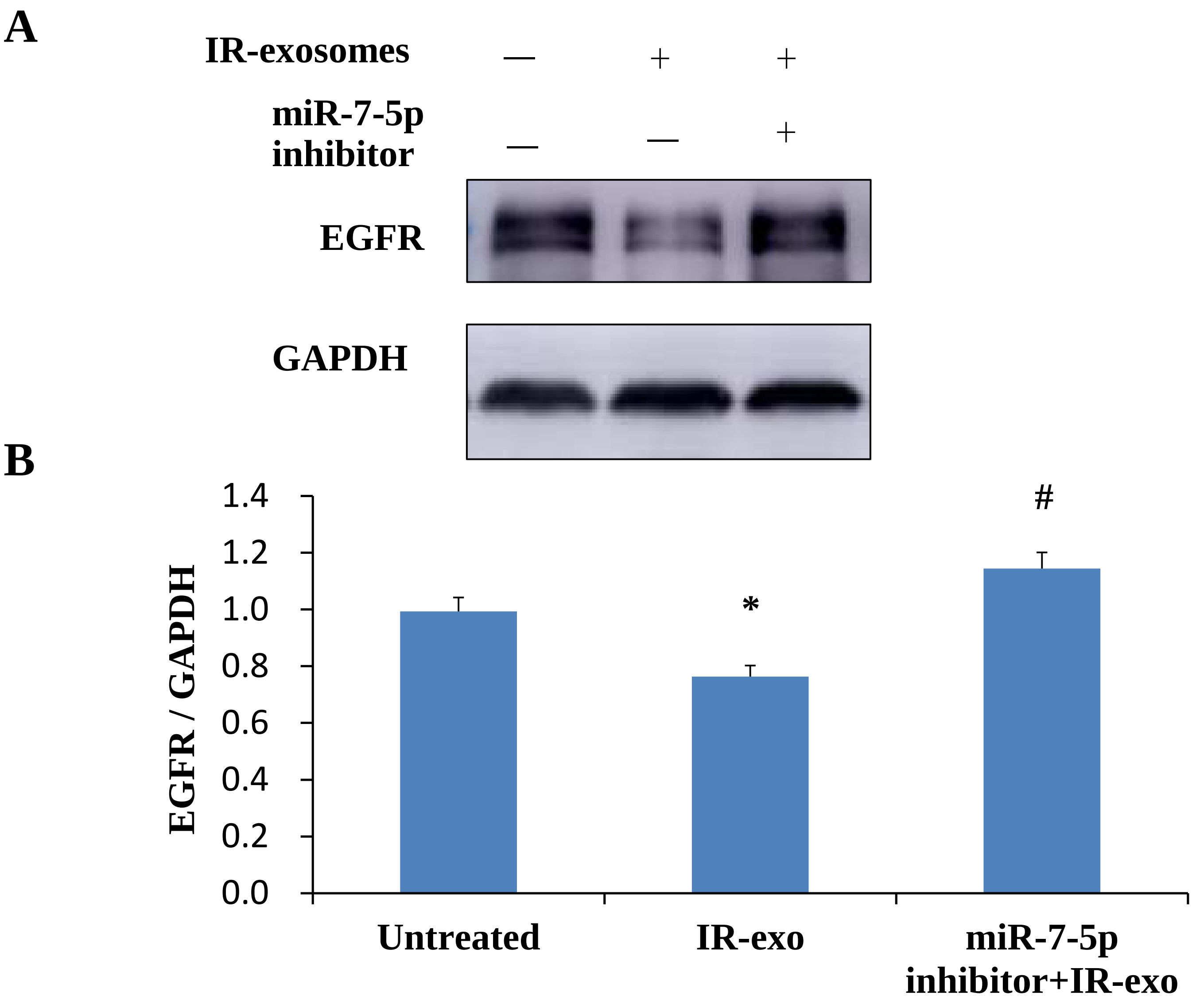

Supplementary Figure Legends

Supplementary Figure 1. The biological pathways analysis of the predicted target genes of IR-induced exosomal miRNAs. The output dataset through the access database (miRFocus) provided target genes and pathway analysis for the miRNA species of interest, an online database that can link genes to its biological pathways through KEGG database. The miRNA-predicted target genes and biological pathways were visualized with Cytoscape software.

Supplementary Figure 2. The effects of miR-7-5p on apoptosis induction and cell cycle distribution of BEP2D cells. A：Detection of apoptosis by annexin V-FITC assay of flow cytometer. B: Detection of apoptosis by colorimetric assay of caspase 3 activity. C: Cell cycle analysis by flow cytometer assay.

Supplementary Figure 3. The effect of conditional medium from 2 Gy irradiated BEP2D cells and exosome-free medium on the proliferation of non-irradiated BEP2D cells.
2 Gy-IR-medium: the conditional medium from 2 Gy irradiated BEP2D cells; 2Gy-IR-medium-exo(-): exosome-free conditional medium. The exosomes were removed from the medium by super-speed centrifugation. Statistical analysis of Bonferroni's post-hoc test was performed. *, $p < 0.05$ as compared with the sample of 2 Gy-IR-medium at the same time point.

Supplementary Figure 4. Exogenous exosomal miR-7-5p regulates EGFR expression in the recipient BEP2D cells. Panel A: EGFR protein levels were detected by western blotting analysis in BEP2D cells after the treatment of exosomes from irradiated BEP2D cells with or without miR7-5p inhibitor. Panel B: Densitometric quantitation of western blotting analysis of EGFR protein expression represented in panel A. *, $p < 0.05$ as compared with the untreated cells. *#, $p < 0.05$ as compared with the IR-exosomes treated cells.
